# Bee Venom and Its Two Main Components—Melittin and Phospholipase A2—As Promising Antiviral Drug Candidates

**DOI:** 10.3390/pathogens12111354

**Published:** 2023-11-15

**Authors:** Carole Yaacoub, Rim Wehbe, Rabih Roufayel, Ziad Fajloun, Bruno Coutard

**Affiliations:** 1 Unité des Virus Emergents, Aix-Marseille University, IRD 190-Inserm 1207, IHU Méditerranée Infection, 13005 Marseille, France; carole.yaacoub@univ-amu.fr; 2 Laboratory of Applied Biotechnology (LBA3B), Azm Center for Research in Biotechnology and Its Applications, Doctoral School for Sciences and Technology, Lebanese University, Tripoli 1300, Lebanon; ziad.fajloun@ul.edu.lb; 3 Biology Department, Faculty of Arts and Sciences, American University of Beirut, Beirut 1107 2020, Lebanon; rimg.wehbe@gmail.com; 4 College of Engineering and Technology, American University of the Middle East, Egaila 54200, Kuwait; rabih.roufayel@aum.edu.kw; 5 Faculty of Sciences III, Department of Biology, Michel Slayman Tripoli Campus, Lebanese University, Tripoli 1352, Lebanon

**Keywords:** viruses, bee venom, melittin, phospholipase A2, antiviral effects, natural extract

## Abstract

Viruses are known to infect most types of organisms. In humans, they can cause several diseases that range from mild to severe. Although many antiviral therapies have been developed, viral infections continue to be a leading cause of morbidity and mortality worldwide. Therefore, the discovery of new and effective antiviral agents is desperately needed. Animal venoms are a rich source of bioactive molecules found in natural goods that have been used since ancient times in alternative medicine to treat a variety of human diseases. Recently, and with the onset of the COVID-19 pandemic, scientists have regained their interest in the possible use of natural products, such as bee venom (BV), as a potential antiviral agent to treat viral infections. BV is known to exert many therapeutic activities such as anti-proliferative, anti-bacterial, and anti-inflammatory effects. However, there is limited discussion of the antiviral activity of BV in the literature. Therefore, this review aims to highlight the antiviral properties of BV and its two primary constituents, melittin (MEL) and phospholipase A2 (PLA2), against a variety of enveloped and non-enveloped viruses. Finally, the innovative strategies used to reduce the toxicity of BV and its two compounds for the development of new antiviral treatments are also considered.

## 1. Introduction

Humans have always valued natural products given their beneficial effects on many pathological conditions [1]. Apitherapy, which is the usage of bee products such as pollen, honey, and propolis as well as bee venom (BV), dates back thousands of years and has been practiced all over the world, mainly in ancient Egypt and Greece [2,3]. In a recent study conducted by Yosri et al. [4], the remarkable antiviral potential of propolis, a resin produced by honeybees, was demonstrated against a multitude of viruses, encompassing adenoviruses, influenza viruses, herpes simplex virus type 1 (HSV-1) and type 2 (HSV-2), human immunodeficiency virus (HIV), and severe acute respiratory syndrome coronavirus 2 (SARS-CoV-2). BV contains a variety of bioactive compounds such as peptides, amines, enzymes, free amino acids, and small molecules [5,6,7]. However, the complete composition of BV has not yet been completely deciphered. So far, at least 214 metabolites have been identified, of which 138 were quantified. Some of the identified low-molecular-weight compounds were carbohydrates, alcohols, and polyols such as pantothenic acid and quinine acid. Other compounds were classified as amines such as histamine and phenylethylamine, as well as modified amino acids (N-acetyl glutamic acid; N-acetyl alanine; N-acetyl aspartic acid; N-methyl aspartic acid). Further peptides of BV, which are present in smaller fractions, include apamin, mast cell-degranulating peptide (MCD), adolapine, secapin, and procamine [8].

Melittin (MEL), a 26-amino acid long peptide that constitutes approximately 40–60% of the dry weight of the venom, serves as the primary component of BV [6]. MEL’s amphipathic nature arises from its hydrophilic carboxyl-terminal region, responsible for its lytic activity, and its hydrophobic N-terminal region, which lacks lytic activity, making it an amphipathic molecule [9]. This amphipathic property enables MEL to disrupt both prokaryotic and eukaryotic cells, as well as natural and synthetic phospholipid bilayers, via the process of pore formation [6,10]. For instance, MEL can disrupt the cell membrane of erythrocytes leading to hemolysis [6,10]. This mode of action induces hormone secretion [11], change of membrane potential [12], and aggregation of membrane proteins [13].

Phospholipase A2 (PLA2), which accounts for around 12–15% of the dry weight of the venom, is the second-most prevalent component in BV [6]. Concerning its primary structure, it is composed of a single polypeptide chain consisting of 128 amino acids, characterized by an active site (-CCxxHDxC-), and a calcium-binding loop. Its secondary and tertiary structure includes two antiparallel disulfide-linked helices in conjunction with the Ca^2+^ binding loop, with five disulfide bridges, the N-terminal helix, and the C-terminal loop that confers the enzyme its flexibility [14].

**Figure 1:**
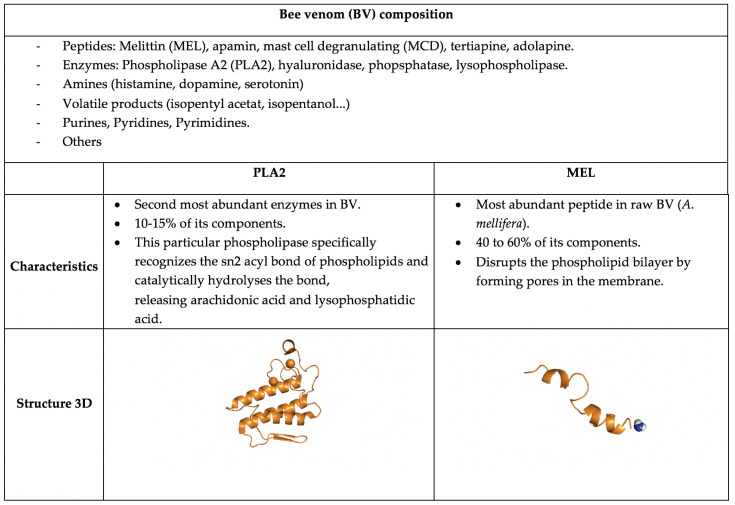
Figure showing BV composition, characteristics, and 3D representation of its two main components, MEL (PDB code 1BH1) and PLA2 (PDB code 1POA).

Figure 1 illustrates the composition of BV, showing the different molecules present in the venom and highlighting its two main components, MEL and PLA2, along with their characteristics and 3D structures.

Arachidonic acid (AA) and lysophospholipids are known to be released as a result of PLA2 hydrolyzing the ester bond of phospholipid membranes at their sn-2 position [15]. The superfamily of PLA2 has been categorized into groups based on various factors, including the requirement for Ca^2+^, the amino acid sequence, and the molecular weight [14]. These categories encompass six main types of PLA2: cytosolic, secretory, calcium-independent, acetyl hydrolase/oxidized lipid lipoprotein related, platelet-activating factor, and lysosomal [14,16]. Bee venom phospholipase A2 (bvPLA2) belongs to the family of secretory PLA2 (group III) with a molecular weight of approximately 15–16 kDa, and its activity is calcium-dependent [17]. Interestingly, its activity can be enhanced by MEL. Indeed, a recent study confirmed the synergistic effect between MEL and bvPLA2, showing that MEL activates bvPLA2 endogenously in intact cells, as well as in in vitro assays [18]. These two biomolecules work synergistically to inhibit the growth of cancer cells [19]. However, the mechanism of action by which MEL activates bvPLA2 is still not clear.

The structural and functional diversity of BV and its bioactive molecules has endowed it with various beneficial effects such as anti-inflammatory, anti-apoptotic, anti-fibrotic, and anti-atherosclerotic effects, which were confirmed by in vitro as well as in vivo evaluations [20]. For instance, Lee et al. [21] demonstrated that crude BV can inhibit adjuvant-induced arthritis, by reducing leukocyte infiltration, preventing erosion of articular cartilage into joints and blocking the development of leukocytosis.

BV can also stimulate the body’s immune system [22]. According to a study by Nam et al. [23], BV increased IFN-γ mRNA expression, enhancing the Th1 cell-dominated immune response. The authors also showed how BV directly affects CD4+ T cell immunological function [23]. Due to its anti-inflammatory and anti-aging properties, BV has been used topically to treat atopic dermatitis (AD) and acne [24]. In an in vivo experiment, the dorsal skin of the mouse model was exposed to BV and/or MEL five times per week for a month (100, 200, and 500 g mixed with normal saline). The trial revealed that BV and MEL reduced skin lesions similar to atopic dermatitis (AD-like) induced by 2,4-dinitrochlorobenzene. Additionally, BV and MEL decreased the expression of chemokines, such as CCL17 and CCL22, and pro-inflammatory cytokines, including IL-1β, IL-6, and IFN-γ, through the blockage of the NF-κB and STAT signaling pathways in an in vitro study using TNF-α/IFN-γ-stimulated human keratinocytes [25]. Clinical studies involving bee venom acupuncture (BVA) on patients with knee osteoarthritis demonstrated the pain-relieving ability of BVA by stimulating aromatase activation in human leukemic cell lines and human osteoblast cells, leading to estrogen production by bone-derived cells, inhibiting the development of osteoarthritis [26,27,28]. Moreover, BV and its main components, MEL and PLA2, have been extensively studied for their anti-proliferative effects [7]. These studies have shown that BV possesses anti-metastatic and anti-invasive properties, which work by inhibiting the expression of MMP-9 and suppressing p38/JNK and NF-ĸβ pathways [7]. BV can also induce apoptosis and/or necrosis of tumor cells by increasing the expression of death receptor 3 (DR3) and inhibiting the NF-ĸβ pathway [29], or by increasing the expression of BAX and CASP3 in rheumatoid synovial fibroblasts [30]. BV apitherapy has also been used to treat various human diseases, including Alzheimer’s disease, rheumatoid arthritis, multiple sclerosis, musculoskeletal pain, intervertebral disc disease, and neuropathic pain [28]. Furthermore, BV has demonstrated antimicrobial effects against a range of microorganisms, including bacteria, fungi, and viruses [31,32].

Viruses rely on the metabolic pathways of their host cells that are used by viruses to complete their life cycle given that they are metabolically inert [33]. They can infect archaea, bacteria, plants, and animals [34]. Ivanovski and Berjerinck discovered the initial virus in the late 1800s [35]. Viruses exhibit a wide range of genome types, including double or single-stranded ones, and can be represented by DNA or RNA [36]. Baltimore proposed a classification system based on the characteristics of viral genomes in 1971 [37]. However, viruses can also be categorized in different ways, such as whether or not the viral particle is surrounded by a lipid bilayer, which is the case of non-enveloped viruses (with no lipid bilayer) or enveloped viruses (with a lipid bilayer, the origin of which varies). Over the past few decades, most newly discovered human pathogens have been identified as viruses [38], including the SARS-CoV-2 coronavirus, which was first detected in Wuhan City, China, in late 2019 and became a global pandemic [39]. Currently, vaccination remains the most effective method to prevent and treat viral diseases [40], but it comes with limitations [41]. Hence, there is a critical need for the development of new approaches to combat viral diseases.

Recent advancements in technology and science, including enhanced analytical tools, genome exploration and engineering, and cell culture techniques, have reignited interest in natural products as potential candidates for drug development. Despite the presence of numerous studies investigating the therapeutic effects of BV and its primary components, the antiviral properties of BV, MEL, and PLA2 have been insufficiently documented in the literature. Therefore, the primary objective of this review is to elucidate the antiviral potential of BV, in conjunction with its two key bioactive components, MEL and PLA2, against a wide range of viruses with varying structural characteristics and genomic compositions.

Taking into consideration the well-established roles of MEL and PLA2 in interacting with lipids, we have structured this review to separately discuss the antiviral effects of BV and MEL on enveloped viruses as well as non-enveloped viruses. Additionally, we provide a concise description of the antiviral activity of bvPLA2, making a comparative reference to snake venom PLA2 (svPLA2). Within this review, we also endeavor to distinguish between the diverse effects exerted by BV and its constituent components. These effects may either entail direct inactivation or destruction of virions, characterized as virucidal actions (such as the disruption of the viral envelope), or indirect interference with various stages of the viral infection cycle, recognized as antiviral actions. It is crucial to acknowledge that these two mechanisms are not always mutually exclusive and that an antiviral compound can exhibit both virucidal and antiviral properties [42,43].

## 2. Effect of BV and MEL against Enveloped Viruses

### 2.1. Enveloped Viruses with Negative-Sense Single-Stranded RNA (ssRNA) Genome

Enveloped negative-sense single-stranded RNA (ssRNA) viruses are termed “negative-sense” viruses due to the fact that their RNA genome is complementary to the mRNA used for translating viral proteins. This implies that prior to the translation by the host cell machinery, the viral RNA is transcribed into a positive-sense RNA [44,45]. Enveloped viruses must fuse their envelope with a host cell membrane to enter the cell and begin replicating [46].

Studies conducted in vitro on BV have demonstrated its virucidal efficacy against H1N1, a member of the influenza A virus family and also known as the swine flu virus, as well as respiratory syncytial virus (RSV), which is a member of the *Paramyxoviridae* family and the genus *Pneumovirus* [47,48,49]. These studies showed that reducing the EC50 (half maximum effective antiviral concentration) of BV to between 1 µg/mL and 2 µg/mL lowered virus infectivity after co-incubating RSV or H1N1 viruses with BV for 30 min prior to inoculating them to the cells. The cytotoxicity of the BV (CC50: half-maximal cytotoxic concentration) was also assessed where it was shown to be between 6 and 8 µg/mL on the cells used in the assay, leading to a low selective index (SI) corresponding to CC50/EC50 between 4 and 5 [49].

In addition to its virucidal effect, BV has shown antiviral activity against other enveloped RNA viruses, such as vesicular stomatitis virus (VSV) [49]. VSV belongs to the family *Rhabdoviridae*, to the genus *Vesiculovirus*, and to the order Mononegavirales. It can be transmitted by arthropods to cattle, horses, and pigs, and can induce fever and the appearance of vesicular lesions in the mouth, tongue, and hoof coronary bands [50,51]. Results showed that BV inhibits the viral infection of VSV in HEK293T cells with an EC50 = 0.5 µg/mL and a CC50 = 8.6 µg/mL, which leads to a SI = 17.22 [49]. To better understand the mechanism of action by which BV inhibits the viral infection, three different treatments were conducted using a GFP-expressing recombinant VSV: BV pretreated group (1): HEK293 cells were pretreated with BV for 12 h at 37 °C then infected with VSV to assess the antiviral effect. BV co-incubation group (2): BV was incubated with VSV for 30 min, then cells were infected with the mixture of BV and VSV (virucidal effect with potential direct effect of BV on VSV particles). Post-treated group (3): first, HEK293 cells were infected with VSV for 30 min, then BV was added (virus replication inhibition after entry). The antiviral effects of BV against VSV were reflected by GFP expression and calculated using the virus titer by standard plaque assay [49]. In parallel, IFN-β levels were measured in HEK293T cell supernatants at the three different times of treatment with BV. In the above-mentioned experiments, BV induced a significant reduction in virus replication at the three different time points of addition compared to untreated cells with BV. Additionally, a higher level of secreted IFN-β was observed when BV was added at the three different time points of addition compared to the control (medium only). The study demonstrated that BV possesses a virucidal activity and can inhibit virus replication after virus entry to the cells by stimulating the type I interferon (INF) signaling pathway [49]. Type I interferon α/β (INFα/β) has shown an antiviral effect against many RNA viruses such as SARS-CoV2 [52]. The presence of IFNβ is crucial to initiate a potent antiviral response, and IFNα alone cannot fully replace its role [53]. The antiviral mechanism induced by BV is thus indirect, as it induces the overexpression of IFNβ, which, in turn, activates the intracellular IFN signaling pathway, particularly the JAK-STAT pathway. Ultimately, this activation can lead to the expression of numerous IFN-stimulated genes (ISGs) with antiviral functions, such as serine/threonine protein kinase, now recognized as PKR [54]

To further understand which BV component is responsible for the antiviral or virucidal effect, the effect of MEL on the same viruses was evaluated as well. MEL showed similar EC50 against VSV, H1N1, and RSV, in the range of 1 µg/mL and SI in the same range (5 to 15) [49]. Interestingly, it was demonstrated that MEL exclusively exerts a virucidal effect unlike BV and does not interfere with the viral infection. Indeed, no significant effect was observed in either scenario: first, when MEL and H1N1 virus were simultaneously added to the cells. Second, when the cells were initially infected with the H1N1 virus, followed by the addition of MEL after a 1 h adsorption period. Subsequently, Uddin et al. confirmed that MEL plays a protective effect on mice against a lethal dose of H1N1 virus and that this protection was primarily attributed to its virucidal effect against H1N1. This was evident as a lethal dose of H1N1 virus was preincubated with MEL [49]. The antiviral action of MEL seems to stem from its direct interaction with the surfaces of viruses. This interaction disrupts the viral structure, effectively inactivating viral particles, a phenomenon referred to as virucidal activity. This disruption prevents the virus from infecting host cells. It was proposed that MEL may bind to the viral surface through surface charge interactions within the virus, reducing viral infectivity, as demonstrated in the case of the scorpion venom peptide variant mucroporin-M1’s virucidal activity against measles, SARS-CoV, and influenza H5N1 viruses [55].

Although the study of Uddin et al. [49] did not include structural studies of the viral particles to investigate the precise nature of MEL’s interaction with viral membranes, some isomeric information regarding the crystal structure of MEL and its known biophysical properties, including α-helical, amphipathic, hydrophobic, and cationic characteristics, discussed that MEL may also interact with the phospholipid bilayer of the viral envelope. Such interaction could potentially lead to changes in lipid organization within the membrane, as reported in cases of hemolysis [56], or the formation of ion-permeable channels, similar to voltage-gated pores [57], or the formation of micellized discs in the membranes, possibly facilitating the diffusion of cellular contents through the created pores [58]. Consistently with the above-described studies, MEL could inhibit the infection of cells by Junin virus (JV) a member of the family *Arenaviridae* in the *Mammarenavirus* genus, a negative-sense single-stranded RNA (ssRNA) enveloped virus that causes Argentine hemorrhagic fever (AHF) disease [59]. The concentration of MEL needed to decrease the virus yield by 50%, was EC50 = 0.86 µM while CC50 was 8.51 µM [60]. Again, despite the low EC50 of MEL, the SI is still low due to its toxicity. However, its mode of action remains undetermined.

### 2.2. Positive-Sense Single-Stranded RNA (ssRNA) Enveloped Viruses

Positive-sense single-stranded RNA (ssRNA) enveloped viruses can be directly translated by the host cells’ machinery to produce viral proteins. Common examples of ssRNA enveloped viruses include Zika virus (ZIKV), dengue virus (DENV), hepatitis C virus (HCV), coronaviruses (CoVs), and human immunodeficiency virus (HIV) [61,62].

BV has been tested on various viruses of the family *Flaviviridae*, including some of the flaviviruses (West Nile Virus or WNV), and hepatitis virus (Hepatitis C virus or HCV). A study done by Ramadan et al. [63] showed that BV exerts a significant virucidal effect against WNV. In fact, when BV was incubated with WNV, a significant decrease in WNV infectivity was observed. However, when Vero cells were co-incubated with BV before infection with WNV, no inhibition of the viral replication was observed [63].

Another study conducted by Sarhan et al. [64] studied the effect of *Apis mellifera* BV against HCV. Results indicated that BV had an inhibitory effect with a low IC50 (0.05 ng/mL) and CC50 in line with other studies (20 μg/mL). BV exhibited direct virucidal activity against HCV, as evidenced by a significant inhibition of HCV infectious particles in culture supernatants following a 2 h pretreatment of the virus with BV. However, no inhibition of virus infectivity was observed when cells were pretreated with BV for 2 h [64]. The anti-HCV effect of BV was neither due to the presence of the main biopeptides such as MEL, apamin, or mast MCD peptide, nor due to the PLA2 activity. The authors hypothesized that a smaller peptide, distinct from the primary biopeptides mentioned, may be responsible for the observed inhibitory effect. Alternatively, it is possible that MEL and PLA2 work synergistically to induce this antiviral effect. MEL has demonstrated virucidal activity against enveloped viruses like Influenza A, as discussed by Sarhan et al. [64]; they explored the virucidal effect of MEL on two distinct enveloped viruses, Influenza A and HCV, attributing the differences to structural and physicochemical disparities in the overall envelope and lipid bilayers of these viruses. Notably, the membrane of HCV originates from the endoplasmic reticulum (ER), whereas the one of influenza A virus is from the plasma membrane [64]. However, they could not rule out the possibility that BV might have an effect on the receptor complexes of host cell components or can interact with components inhibiting viral entry [64].

MEL has also shown an antiviral effect against HIV and so far, at least two mechanisms of action of MEL have been described. MEL-lipid nanoparticles act as fusogenic and pore-forming toxins [65]. Researchers suggest that HIV-1 particles connect with MEL nanoparticles through a traditional process called lipid-to-lipid membrane hemifusion. This is similar to what happens between the layers of liposome membranes and MEL nanoparticle layers. This mechanism makes it much easier for MEL to move from the lipid layers of nanoparticles to the envelope layers of HIV-1. Once there, MEL can clump together and create pores, deactivating the virus effectively [66]. Additionally, compared to what Hood et al. [66] demonstrated, Wachinger et al. [67] revealed a different mechanism. They specifically demonstrated that the amphipathic helical region (amino acids 1–20) of MEL is responsible for the suppression of HIV-1 caused by MEL. Instead of a direct impact on the cell membrane, the authors hypothesize that inhibition results from the disruption of intracellular processes involved in the production of HIV protein [67]. The proposed mechanism of action is supported by a couple of observations. First, MEL is easily taken up by the cells, so it does not have time to interact with the virus. Second, a derivative of MEL lacking the basic carboxy-terminal hexapeptide—a component essential for its lytic activity—exhibits a similar inhibitory impact on HIV-1, although at slightly higher concentrations [67]. Another study demonstrated that MEL suppresses intracellular production of HIV proteins by the reduction in overall levels of HIV-1 mRNAs in a dose-dependent manner, suggesting a decrease in HIV long terminal repeat (LTR) activity, with IC50 values in the range of 0.9–1.5 µM [68].

Additionally, a recent study utilized the anti-inflammatory drug Sitaglipsin (SIT). For the treatment of adult patients with type 2 diabetes (T2D), SIT, a dipeptidyl peptidase-4 inhibitor, is approved in more than 130 countries throughout the world as monotherapy and in combination with other anti-hyperglycemic drugs [69], along with MEL against the SARS-CoV-2 virus. Results showed that the complex SIT-MEL has a potent antiviral effect against the SARS-CoV-2 virus with IC50 = 8.43 µM [70]. In addition, the delivery and the uptake of the optimized formulation SIT-MEL were enhanced which yielded a greater antiviral activity against SARS-CoV-2 [70].

### 2.3. Enveloped DNA Viruses

Herpes simplex virus (HSV), a member of the *Herpesviridae* family of viruses with double-stranded linear DNA enclosed by a capsid and an envelope, has been shown to be susceptible to the virucidal effects of BV [71]. The virucidal effect of BV on HSV was significant, with an EC50 of 1.52 µg/mL and CC50 of 7.13 µg/mL, leading to SI = 4.69 [49]. Meanwhile, MEL, on its own, was able to inhibit viral replication in Vero cells, likely by inhibiting the attachment of HSV to cells in a dose-dependent manner [72]. MEL predominantly inhibited virus attachment, and to a lesser extent, virus penetration, suggesting its potential interaction with calmodulin-like domains on the viral envelope involved in virus attachment [72].

## 3. Effect of BV and MEL against Non-Enveloped Viruses

Naked viruses, sometimes referred to as non-enveloped viruses, are viruses that lack a lipid envelope covering their capsid. Non-enveloped human viruses are likely to interact with glycosaminoglycans (GAGs) during cell entry, although the structural details of the interactions between GAGs and viral capsid proteins are not well understood [73]. Several studies have compared BV and MEL to various non-enveloped virus families, including picornaviruses, adenoviruses, and the human papillomavirus (HPV).

BV and MEL exhibit virucidal effects against coxsackievirus B3 (CV-B3) and enterovirus-A71 (EV-71), even though these viral particles lack a lipid envelope [49]. These two viruses belong to the *Picornaviridae* family, which includes non-enveloped viruses with a single-stranded positive-sense RNA genome. CV-B3 is responsible for many diseases like myocarditis and pancreatitis in young children [74], while the EV-71 virus causes hand, foot, and mouth disease (HFMD) and neurological issues in children between 5 to 7 years old due to their weaker immune system [75]. EV-71 symptoms include a severe rash in the hand, foot, and mouth areas resembling blisters, fever, and painful sores [76]. A study conducted by Uddin et al. [49] demonstrated the virucidal effect of BV and MEL against CV-B3 and EV-71, with EC50 of 0.5 µg/mL and 0.49 µg/mL, respectively, for BV and 0.99 µg/mL and 0.76 µg/mL, respectively, for MEL. Furthermore, the results demonstrated that MEL may prevent viral infection even when it only comes into contact with the virus for a brief moment. While MEL inhibits viral replication, it does not affect the initial stages of the viral life cycle, suggesting that MEL acts directly on the virus. Given that the studies suggest that the mode of action by which MEL exerts its virucidal effect is based on its interaction with the membrane of enveloped viruses, its virucidal effect against non-enveloped viruses is obviously via an alternative mechanism of action that needs further investigation.

When incubated with the virus prior to cell infection, BV reduces adenovirus-7 infectivity in addition to picornaviruses. However, no significant effect was observed when cells were exposed to BV for 6 or 24 h prior to infection [63]. This finding highlights the virucidal potential of BV rather than its antiviral effect against adenovirus. In addition, BV inhibits the growth of cervical cancer cells infected by HPV by the downregulating of E6/E7 oncoproteins, which are essential for immortalization and transformation of human squamous epithelial cells [77].

Table 1 represents the antiviral effect of BV against enveloped and non-enveloped viruses, providing information on the half-maximal effect concentration (EC50), and the mechanism of action. Similarly, Table 2 represents the antiviral effects of MEL against enveloped and non-enveloped viruses with the EC50 as well as its mechanisms of action.

## 4. In Vitro Antiviral Effect of bvPLA2

bvPLA2 is the second-most prominent biomolecule in the BV. The vast majority of studies conducted on bvPLA2 have focused on its anti-inflammatory, anti-tumor, and anti-bacterial effects [17]. However, its antiviral effect has been poorly mentioned in the literature. For example, the virucidal activity of bvPLA2 against HCV, DENV, and JEV was reported, with IC50 of 117, 183, and 49 ng/mL, respectively, while no cytotoxic or hemolytic activity was observed even at high concentrations (10 µg/mL) [78]. Nonetheless, the most in-depth studies conducted on bvPLA2 depicted its antiviral mode of action, independent of its catalytic activity [79,80]. In their initial study, the authors explored the impact of secreted PLA2 and identified that bvPLA2 could inhibit the viral replication of HIV-1. Importantly, the inhibition of the viral infection occurred at the virus entry stage and was not due to a direct interaction with the particles. Building upon this discovery, Fenard et al. [80] synthesized 12 bvPLA2-derived peptides. Among these peptides, only one, from amino acids 21 to 35, exhibited the ability to inhibit the replication of HIV-1 with an IC50 of 2 µM. In addition, the researchers showed that this peptide may prevent the CXCR4 receptor, one of HIV’s co-receptors, from binding to a natural ligand. This finding suggests that by binding to one of the viral receptors, the receptor may block the entry of the virus by competing with the viral particle. Given that bvPLA2 does not compete with the viral particle on CRCX4, it is noteworthy that the mechanism of action of this peptide is different from that of bvPLA2 [80].

The antiviral activity of PLA2 has only been reported for some of its structural and functional analogs isolated from snake venoms, with a wide range of efficacy on a broad spectrum of viruses (Table 3 for details) [78,81,82,83].

## 5. Innovative Strategies Used to Reduce the Toxicity of BV and MEL

Despite the therapeutic utility of BV, its safety profile remains an essential limiting concern. Therefore, many studies are focusing on developing new strategies that can reduce the cytotoxicity of BV and enhance its efficacy.

A study conducted by Lee et al., 2021 [84] showed that the detoxification of BV, achieved by hydrolyzing melittin and removing other components, significantly increases its anti-inflammatory activity. This detoxified BV also exhibits reduced cytotoxicity and allergenic activity when compared to the original BV. Furthermore, when compared to untreated BV, detoxified BV significantly inhibits mRNA expression levels of pro-inflammatory cytokines such as TNF-α, IL-6, and iNOS. Moreover, it effectively inhibits the phosphorylation of IκBα in RAW 264.7 cells and induces degranulation in RBL-2H3 cells. It is important to mention that the authors confirmed that the pharmacological effectiveness of the detoxified BV was conserved. 

Another recent strategy that was adopted to reduce the toxic effect of BV in therapeutics like prostate cancer was the development of cross-linked chitosan enteric-coated microspheres as a controlled drug carrier system for the effective delivery of oral BV. These microspheres are microparticles composed of biodegradable polymers like chitosan. The study showed that free BV was more potent against the growth of human prostate adenocarcinoma (PC3) cells followed by optimized microspheres than doxorubicin, a type of chemotherapy drug. Additionally, the optimized microsphere formula induced apoptosis and reduced necrosis at effective concentrations. Also, microspheres did not affect the viability of normal oral epithelial cells [85].

As previously mentioned, the main component of BV, MEL, is the compound with the most abundant pharmacological properties. Nevertheless, the clinical application of melittin is limited due to its serious hemolytic and cytotoxic effects. The strategies adopted to properly deliver MEL work somehow in the same way. These delivery system approaches aim to (1) increase the delivery efficiency of MEL, (2) mask MEL to prevent it from interacting with cell membranes, as well as (3) hide its positive charge so it does not bind to other proteins in vivo [86]. There are mainly two strategies to overcome the toxic effects of MEL: (a) Modified MEL and conjugates by changing its amino acid sequence or linking it with other polypeptides with other properties, (b) nano-drug delivery vehicles such as polymer, lipid, inorganic carriers, etc. For example, the hemolytic activity of MEL-based nanoparticles was significantly reduced when compared to that of the native MEL. Another strategy that has been recently adopted is the phosphorylation of MEL in 10Thr and 18Ser residues where it has been shown that the phosphorylated form of MEL has a lower allergenic response than that of native MEL. Moreover, adding DapAMCA (Trp19 substitution with non-canonical fluorescent amino acid) residue to melittin modified its mechanism of action with the cell membrane, yielding a reduced hemolytic toxicity and an increased selectivity index, with an up to a fivefold increase in comparison to melittin, as per in vitro anticancer activity and hemolytic studies [87]. The preservation of antiviral properties together with the reduction in its toxicity have been investigated for MEL. For instance, Falco et al. [88] showed that incorporation of MEL into immunoliposomes containing antibodies against viral glycoprotein enhances therapeutic targeting and reduces the MEL dose, enhancing the SI. Using nanoparticles to create MEL-based virucidal formulations against HIV-1 has also been shown by L. Hood et al. [66] to reduce the toxicity of MEL. Peptides derived from MEL can be used as a non-toxic alternative for MEL [67], similar to how it has been carried out for bvPLA2 [80]. 

## 6. Conclusions

**Figure 2:**
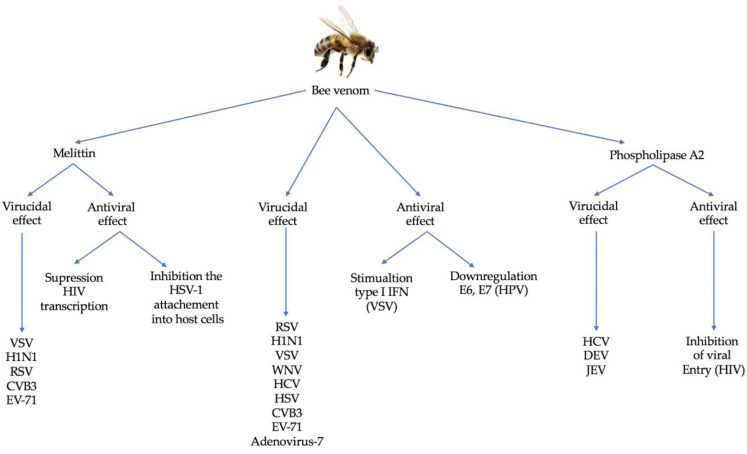
Different mechanisms of action of BV against different families of viruses.

Many chronic human ailments have been treated with BV for thousands of years. This review highlights the antiviral effects of BV and its primary components, MEL and PLA2, and how they act on enveloped and non-enveloped viruses. By serving as virucidal molecules or inhibitors of viral infection/replication, BV and MEL display moderate to considerable antiviral action, often in the range of µg/mL, against a wide range of viruses. There are many mechanisms of action by which BV and MEL act on viruses. For example, they may directly interact with the viral envelope or capsid proteins and alter how viruses interact with their hosts, or they may indirectly reduce viral replication by inducing type I IFN signaling. The safety profile of both BV and MEL remains an essential limiting concern and many innovative strategies such as nano-drug delivery vehicles are being developed to reduce their intrinsic toxicity. The many antiviral effects of BV on viruses are schematized in Figure 2, which also shows the various kinds of these susceptible viruses. Finally, the clinical application of BV and its components as antiviral drugs is still a long process ahead. Nonetheless, extensive work focusing on the use of natural products in therapeutics as well as the advancement of nanomedicine will allow BV, as well as MEL and bvPLA2, to be considered as definitive future antiviral drug candidates.

## Figures and Tables

**Table 1:** Description of the different types of enveloped and non-enveloped viruses sensible to the BV, the different EC50 and SI values obtained, as well as the mechanism of action exerted by the BV on these viruses.

		Viruses	EC_50_	SI	Mechanism of Action	References
**Bee venom’s antiviral effect**	**Enveloped viruses**	Respiratory Syncytial Virus (RSV)	1.17 µg/mL	5.34	Virucidal effect	[49]
		Influenza A (H1N1)	1.81 µg/mL	4.61	Virucidal effect	[49]
		Vesicular Stomatitis Virus (VSV)	0.5 µg/mL	17.22	Inhibition of virus replication after virus enters the cells. Stimulating type I IFN signaling. Virucidal effect	[49]
		West Nile Virus (WNV)			Virucidal activity	[63]
		Human Hepatitis C Virus (HCV)	0.05 ng/mL	400,000	Direct virucidal activity. Probability to have effect on the entry of the virus in the cells.	[64]
		Herpes Simplex Virus (HSV)	1.52 µg/mL	4.69	Virucidal effect	[49]
	**Non-enveloped viruses**	Coxsackievirus B3 (CVB3)	0.5 µg/mL	17.96	Virucidal effect	[49]
		Enterovirus (EV-71)	0.49 µg/mL	18.3	Virucidal effect Decrease VP1 mRNA expression.	[49]
		Human Papillomavirus (HPV)			Downregulation of E6/E7 protein of HPV	[77]
		Adenovirus type-7			Virucidal activity	[63]

**Table 2:** Description of the different types of enveloped and non-enveloped viruses sensible to the MEL, the different EC50 and SI values obtained, as well as the mechanism of action exerted by the MEL on these viruses.

		Viruses	EC_50_	SI	Mechanism of Action	References
**MEL’s antiviral effect**	**Enveloped viruses**	Vesicular Stomatitis Virus (VSV)	1.18 µg/mL	5.27	Virucidal activity	[49]
		Influenza Virus (H1N1)	1.15 µg/mL	6.66	In vivo, protect mice from lethal dose of H1N1(virucidal effect)	[49]
		Human Respiratory Syncytial Virus (RSV)	0.35 µg/mL	14.34	Virucidal effect	[49]
		Junin Virus (JV)			Antiviral activity (mechanism not dermine)	[60]
		HIV	0.9–1.5 µM		· Direct effect on the virus. · Disruption of intracellular processes involved in HIV protein production. · Suppressing intracellular production of HIV structural proteins, by reduction in overall levels of HIV-1 mRNAs in a dose-dependent manner, suggesting a reduction in HIV long terminal repeat (LTR).	[66,67,68]
		SARS-CoV-2 (SIT-MEL)	8.43 µM			[70]
		Herpes Simplex Virus (HSV)	0.5 µM		Inhibiting the attachment of HSV-1 into hot cells by inhibiting the Na+, K+ pump leading to the inhibition of the cell fusion.	[72]
	**Non-enveloped** **viruses**	Enterovirus 71 (EV-71)	0.76 µg/mL	5.75	Decreasing four times the mRNA expression levels of capsid protein VP1 in EV-71-infected cells compared to untreated cells.	[49]
		Coxsackievirus H3	0.99 µg/mL	4.40	Virucidal activity	[49]

**Table 3:** Representation of the antiviral effect of bvPLA2 *versus* svPLA2 against enveloped and non-enveloped viruses, as well as their mechanism of action.

bvPLA2		svPLA2	
Viruses	Mode of Action	Viruses	Mode of Action
HIV-1	Blocking the virus entry [79,80]	HCV	· Virucidal effect · Downregulating viral receptors or neutralization of the infectivity of the virus when released into the medium during viral inoculation [78]
		Dengue virus (DENV)	
		Japanese encephalititis virus (JEV)	
		YFV and DENV	Virucidal effect [82]
		Rocio virus Oropouche virus Mayaro virus	Virucidal effect [81]
HCV, DENV, and JEV	Virucidal effect [78]	Chikungunya virus	Inhibition of viral entry into cells [83]

## Data Availability

Not applicable.
